# Enhancing the production of volatile fatty acids by potassium permanganate from wasted sewage sludge: A batch test experiment

**DOI:** 10.1016/j.heliyon.2023.e21957

**Published:** 2023-11-07

**Authors:** Antonio Mineo, Alida Cosenza, Bing-Jie Ni, Giorgio Mannina

**Affiliations:** a Engineering Department, Palermo University, Viale delle Scienze ed. 8, 90128, Palermo, Italy; b Centre for Technology in Water and Wastewater, School of Civil and Environmental Engineering, University of Technology Sydney, Sydney, NSW 2007, Australia

**Keywords:** Acidogenic fermentation, Bio solid management, Circular economy in the water sector, Potassium permanganate, Waste activated sludge

## Abstract

Recovering resources from wastewater treatment is vital for the transition from a linear to a circular economy model in the water sector. Volatile Fatty Acids (VFAs) are valuable products among the possible recovered resources. This study investigates the influence of potassium permanganate (KMnO_4_) addition during acidogenic fermentation of waste activated sludge for enhancing VFAs production. Specifically, different fermentation batch tests with and without KMnO_4_ addition were carried out using two distinctive sewage sludges as feedstocks. Results showed that KMnO_4_ addition increased the VFAs yield up to 144 and 196 mgCOD/g VSS for the two sludges. When KMnO_4_ was used as pre-treatment, 55 % of sCOD were VFAs. This latter result was mainly debited to the recalcitrant organics’ disruption promoted by the oxidative permanganate ability.

## 1 Introduction

Sewage sludge (SS) management is one of the most critical issues in wastewater treatment plant (WWTP) operation [1]. Overall SS management accounts for over 50 % of the WWTP operating costs. Therefore, new technologies and strategies are required to reduce the SS quantity and increase its capacity to extract valuable materials according to the water resource recovery facility concept [2]. Anaerobic digestion has been widely used to reduce the amount of SS and to recover energy via biogas [3]. Methane (CH_4_) is one of the main gases produced by anaerobic digestion. However, despite CH_4_ production allows to produce energy it has the inconvenient of increasing direct greenhouse emissions that still needs to be solved [4]. Nonetheless, other high-value products can be obtained from SS by using microbial communities’ activity which seems a promising alternative to conventional sludge disposal as landfilling or incineration [5]. Substances like volatile fatty acids (VFAs), obtained through anaerobic digestion [6], can be used as substrates for polyhydroxyalkanoate (PHA) production adopted by mixed microbial communities (MMC). Recently, many efforts have been made to integrate PHA production in conventional WWTPs so that the goal of the process is to obtain renewable products (i.e. PHA) for a sustainable future [7,8].

Despite its rising potential, VFAs production from SS anaerobic digestion still needs to be optimised [9]. Several studies have focused on the influence of operation variables on the process [10] and their optimisation to maximise VFAs yield and productivity [11,12]. Poor sludge solubilisation and low VFAs production are the main challenges that still need to be overcome to achieve a feasible process [13].

In this context, many studies reported that alkaline pH enhances the disruption of the sludge flocs structure, thus providing more substrate for the acidogenic step, and preventing the methanogens activity [[14], [15], [16]]. Usually, advanced oxidation processes are used to achieve alkaline conditions and exploit oxidative abilities of chemicals such as sodium or calcium hydroxide (NaOH, Ca(OH)_2_). Recently, Xu et al. [11] have investigated the adoption of a robust green oxidant such as potassium permanganate (KMnO_4_) given the improving VFAs production and fermented effluent quality. Adding KMnO_4_ increases sludge pH sharply to generate an alkaline environment during the first few days, which later evolves into an almost neutral pH value, generally at 6.8. The oxidant effect may enhance the organic matter disruption and product solubilisations in the first days of experimentation thanks to the high pH value [17]. As the tests follow, a more acidic environment would promote the acidogenic step thus overall enhancing the whole process yield [18,19]. Recently, Wang et al. [20] applied urea hydrogen peroxide (UHP) pretreatment on sludge acidogenic fermentation. UHP effect and mechanisms are comparable to those reported for KMnO_4_: the established alkaline conditions, coupled with the free radicals generated, disrupted the organics in the sludge, thus increasing VFAs production up to 8800.9 mg COD L^−1^. Sheng et al. [21] evaluated the influence of calcium peroxide in waste activated sludge acidogenic fermentation. Overall, calcium peroxide enhanced VFAs production up to 7471-7 mg COD/L, which was 1.5 times higher than the control test. Also, calcium peroxide has been widely studied in SS fermentation, proving its effectiveness both in chemical (increasing the organics release) and biological processes (increasing enzymes activity related to the hydrolysis and the VFAs biosynthesis) [22].

Despite studies on the production of VFAs from sewage sludge have been carried out so far, the addition of KMnO_4_ in the process is relatively new, despite its economic and environmental benefit [23,24]. Still, the oxidant efficiency has been widely proved but not correlated with the different SS features. To fill the above gaps, this study aims to gain insights into the effect of KMnO_4_ in SS fermentation, evaluating the impact on organic matter hydrolysis and acidogenesis. SS fermentation by the addition of KMnO_4_ was investigated in batch tests. Two different WWTP sources were used to collect the SS to investigate how the oxidant can interact with varying features of sludge.

## 2 Materials and methods

### 2.1 Batch fermentation reactor

**Fig. 1:**
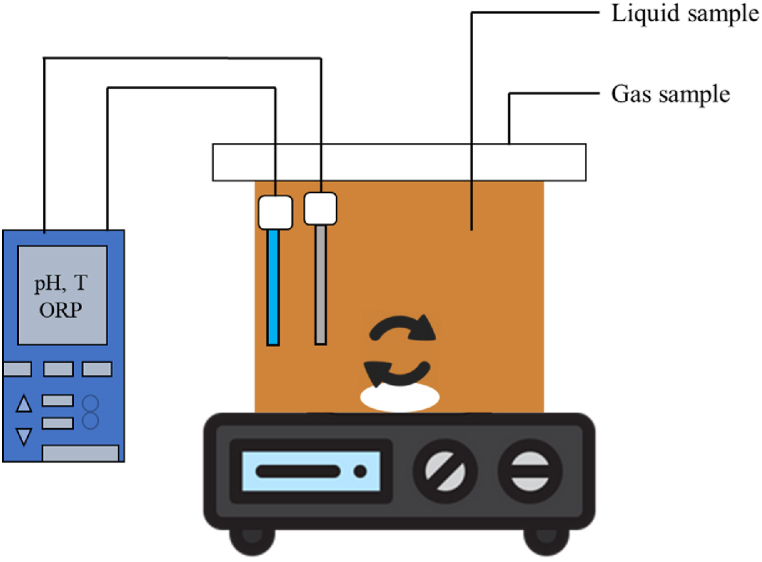
Batch fermentation test set up.

Bench-scale batch fermentation tests have been performed in 1100 mL of magnetic stirred glass bottles (Fig. 1). The bottles are closed with a cover equipped with two sampling ports for liquid and gas sampling. The bottles are connected to a WiFi - Multi 3630 IDS “WTW” (Xylem brand) probes for continuously monitoring the operating parameters (such as temperature, pH …).

### 2.2 Wastewater treatment plant, sewage sludge and wastewater features

Two sludge samples were taken from the pilot plant at the Water Resource Recovery Facility of Palermo University [2] and from Marineo (Italy) WWTP [25]. Table 1 summarises pilot (Plant A) and real plant (Plant B) features.

**Table 1:** Pilot plant and WWTP features.

Parameters	Symbol	Unit	Plant A	Plant B
Flow rate	Q	m^3^/h	0.48	90
Sludge retention time	SRT	day	27	20
Food to microorganism	F/M	kg BOD/kg TSS x day	0.26	0.16

Plant A is composed of a wastewater treatment line based on a conventional activated sludge system, conceived for carbon and nitrogen removal, and a sludge line conceived to produce PHA from wastewater. Plant B has a CAS system. Table 2 summarises the features of the adopted SS for the fermentation tests.

**Table 2:** Sewage sludge features.

Parameters	Plant A	Plant B
pH	7.51	7.32
Total Suspended solids, TSS (g/L)	4.70	4.85
Volatile Suspended Solids, VSS (g/L)	3.90	2.88
Total Chemical Oxygen Demands, TCOD (mg/L)	5000	3300
Soluble Chemical Oxygen Demands, sCOD (mg/L)	106	59
Ammonium, NH_4_^+^-N (mg/L)	4.70	2.30
Phosphate, PO_4_^3-^-P (mg/L)	2.32	3.60
Proteins EPS (mg/g VSS)	30.04	45.03
Carbohydrate EPS (mg/g VSS)	3.48	10.08

### 2.3 Experimental campaign

The experimental campaign was performed using 4 batch tests, namely T1- T4 (Table 3). Specifically, potassium permanganate (KMnO_4_) was used as an oxidant for T2 and T4 at a concentration of 0.1 g KMnO_4_/TSS [11], while T1 and T3 were the reference tests without the chemical addition.

**Table 3:** Details of the batch fermentation tests.

Batch test	TSS (g/L)	Details	Source
T1	4.7	Reference	Plant A
T2	4.7	0.1 g KMnO4/g TSS	Plant A
T3	4.8	Reference	Plant B
T4	4.8	0.1 g KMnO4/g TSS	Plant B

The tests were carried out for 14 days during which sCOD, VFAs, NH_4_^+^-N and PO_4_^3-^-P concentration were analysed. Temperature, pH and Oxidation Reduction Potential (ORP) have been monitored using a WiFi - Multi 3630 IDS “WTW and related probes.

### 2.4 Analytical methods

sCOD, TCOD, VSS, TSS, NH4–N and PO4–P concentrations were analysed according to standard methods suggested by APHA [26]. Extracellular polymeric substances (EPS) and soluble microbial products (SMP) extraction and analysis method have been performed according to Le-Clech et al. procedure [27]. EPS and SMP extracted samples are measured at 700 nm wavelength for proteins and 625 nm for carbohydrates in a UV-VIS spectrophotometer (UVmini-1240, Shimadzu, Japan). The VFAs measuring was performed by using gas chromatography (GC) after the extraction with dimethyl carbonate (DMC-OEI) as reported by Ghidotti et al. [28]. An Agilent Technologies 7820A GC with a flameionisation detector (FID) and a DB FFAA column (30 m ×0.25 x mm x 0.25 μm) was used to analyse VFAs samples following the GC protocol described by Montiel-Jarillo et al. [29]. Formic, acetic, propionic, isobutyric, butyric, isovaleric, valeric, isocaproic, hexanoic and n-heptanoic acids were analysed. VFAs concentration (mg/L) was converted into COD concentration (mgCOD/L) by using the conversion factors [30].

Over the experimental period, COD solubilisation was calculated according to equation (1), proposed by Mohammad Mirsoleimani Azizi et al. [31].

(1) $$COD\;solubilization=\frac{sCOD_{t}-SCOD_{0}}{TCOD_{0}}$$

Where (t) and (0) refer to the generic and initial time, respectively. Sporadic carbon dioxide (CO_2_) and CH_4_ dissolved and off-gas measurements have been performed using the same GC.

## 3 Results and discussion

### 3.1 Effect of KMnO_4_ dosage on sludge hydrolysis

#### 3.1.1 sCOD and nutrients concentrations

**Fig. 2:**
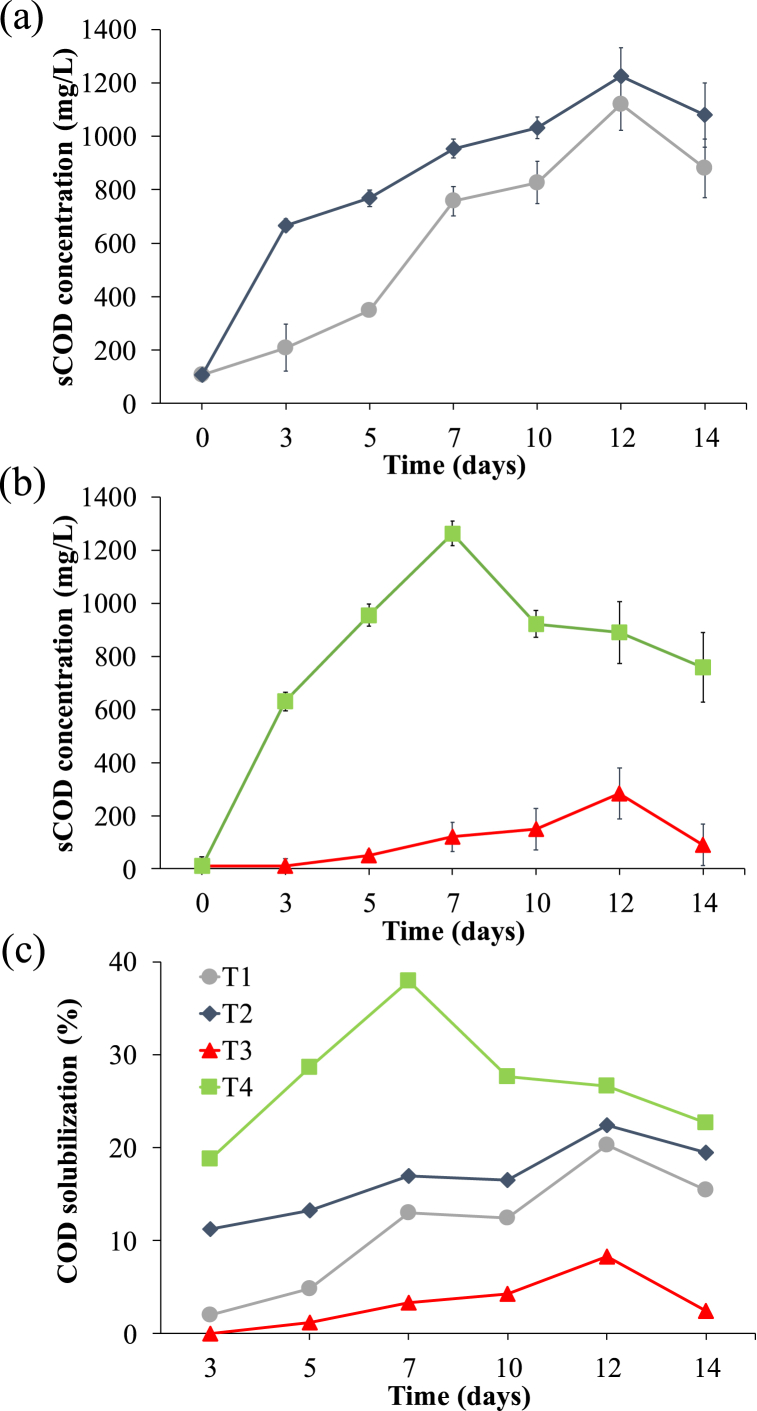
The trend of sCOD concentration during T1-T2 (a) and T3-T4 (b) and COD solubilisation (c).

Results reported in Fig. 2 show the trend of soluble COD and COD solubilisation for all the tests. The sCOD production was enhanced by the KMnO_4_ addition both in T2 and T4 (1226 and 1263 mg COD/L on the 12th and 7th day, respectively) while it achieved the peak value of 1119 and 284 mg COD/L on the 12th day for T1 and T3, respectively. Adding the oxidant to sludge from plant A resulted in a slight increase in the sCOD concentration at the peak day (+9.6 %) while it determined a much higher increase when plant B's sludge was tested (+344 %). Also, T4 reached the sCOD production peak 5 days before T2 due to the hydrolysis enhancement [32]. This can be related to the different initial EPS concentrations of the sludge used (Table 2), since the main oxidant effect is the organics matter disruption [33]. Despite the mere difference in EPS concentrations, the EPS fraction quality may have determined a crucial role in the fermentation process. Indeed, despite the lower EPS concentration for T1, the fermentation reached a much higher sCOD concentration than T3. This underlines how the EPS fraction in T1-T2 was easily dissociated even without the oxidant addition while it was much more resistant to normal acidogenic fermentation conditions in T3-T4 [34]. COD solubilisation (Fig. 2c) follows the same trend discussed before: KMnO_4_ enhanced the disruption of complex organic matter and its effect was highly noticeable when the sludge from the full scale WWTP was used. The oxidant addition resulted in a COD solubilisation increase of almost 30 % for plant's B sludge, while it was only 2 % for T2. Despite the difference in EPS concentration and share, recalcitrant organics may have played a crucial role in the process. Despite having a higher EPS content, sludge B had a lower VSS/TSS ratio compared to sludge A. This may suggest that the sludge is rich in recalcitrant organics which are more resistant to hydrolysis, reason why the oxidant addition effect was far more noticeable in T4 than T2.

**Fig. 3:**
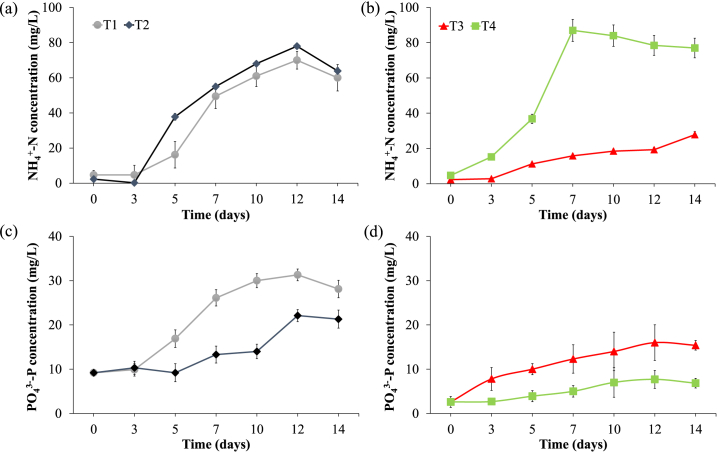
Trends of ammonium and phosphate concentration during T1-T2 (a,c) and T3-T4 (b,d).

Fig. 3 a-b shows the NH_4_^+^ during the experiments. T2 and T4 enhanced NH_4_^+^ dissolution in the supernatant due to protein hydrolysis [35,36]. The higher protein degradable content in T4 is also shown in the higher ammonium concentrations at the peak day than in T2 (87 and 78 mg/L respectively). No significant difference was found between T1 and T2 at the peak day (70 and 78 mg/L respectively), following the same trend reported for sCOD. PO_4_^3−^ concentration trends are reported in Fig. 3 c-d. When the KMnO_4_ is added, the consequently alkaline condition tends to promote phosphate precipitation thus reducing its concentration in the supernatant. For this reason, T1 and T3 showed higher phosphate concentrations at the peak day (31 and 16 mg/L respectively) than T2 and T4 (22.1 and 5 mg/L respectively).

#### 3.1.2 Production of protein and carbohydrates

**Fig. 4:**
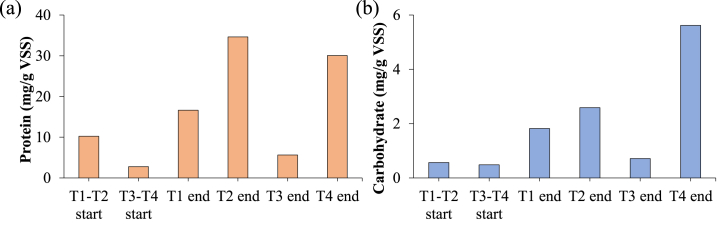
SMP proteins (a) and carbohydrates (b) concentrations at the start and end of the fermentation tests.

Fig. 4 reports the soluble proteins (Fig. 4a) and carbohydrates (Fig. 4b) concentration at the start and end of the experiments. T2 and T4 sludge had the highest protein production (34.6 and 30.1 mg/g VSS respectively) with a slight difference which was much more evident for carbohydrates production. Indeed, T4 carbohydrates concentration almost doubled T2 (5.62 and 2.59 mg/g VSS respectively). Despite being low amounts, carbohydrates may have played a crucial role in the fermentation and more specifically in the KMnO_4_ action. T1 protein and carbohydrate production was around threefold the T3 concentration (16.63, 1.82 and 5.63 and 0.71 mg/g VSS for protein and carbohydrate in T1 and T3 respectively). As stated before, the oxidant addition enhanced the organic matter disruption, as demonstrated by the increase in soluble protein and carbohydrates concentrations at the end of the fermentation [37].

### 3.2 Effect of KMnO_4_ dosage on sludge acidogenesis

**Fig. 5:**
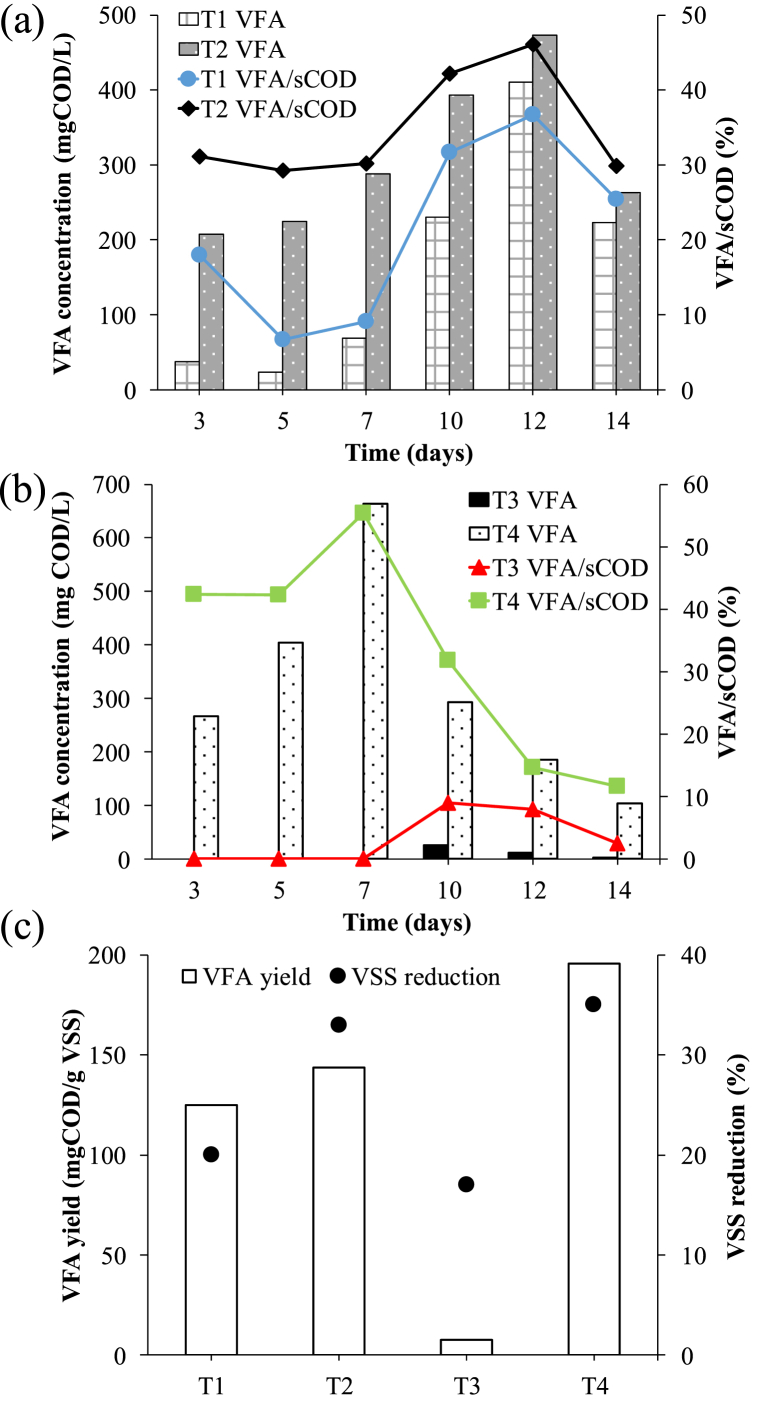
VFAs concentration and VFAs/sCOD ratio for T1-T2 (a) and T3-T4 (b) and VFAs yields and VSS reduction (c).

The trend of VFAs production was similar to sCOD for all the tests (Fig. 5 a-c). In T1-T2 the VFAs production was slightly increased from 125 to 144 mg COD/g VSS, respectively. Also, in the VFAs production the effect of KMnO_4_ was highly noticeable in T3-T4 where it increased the VFAs concentration (7 and 196 mg COD/g VSS, respectively). Overall, the highest total VFAs content (664 mg COD/L) in T4 was achieved on day 7 and accounted for about 55 % of sCOD, while the peak value for T2 (day 12, 474 mg COD/L) accounted for about 38 % of sCOD. Finally, both T2 and T4 achieved the highest VSS reduction (33 and 35 % respectively). These results prove the KMnO_4_ efficiency both in hydrolysis and acidogenic step of the fermentation: for T4 it increased sCOD production (+344 %), it enhanced the COD solubilisation (+30 %) and increased the VFA yield up to 196 mg COD/g VSS compared to the control test (7 mg COD/g VSS). These results also prove that potassium permanganate efficiency is related to the sludge features, since it was much more effective for a sludge rich in recalcitrant organics [38].

**Fig. 6:**
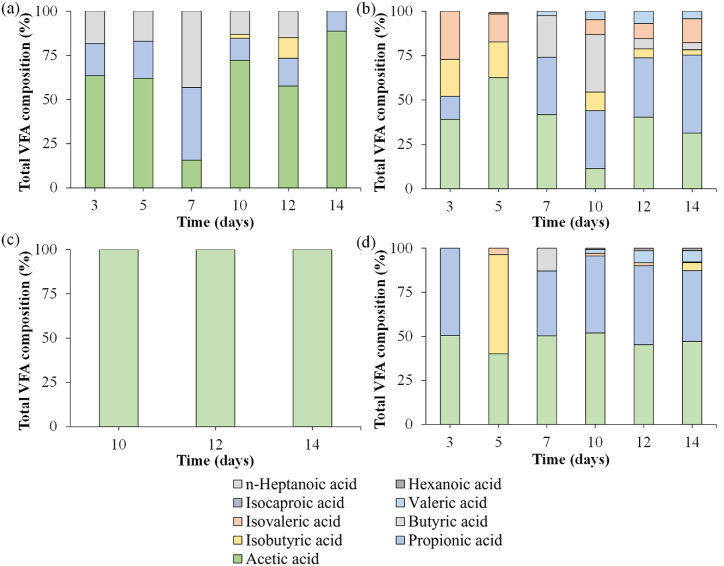
VFAs composition for T1 (a), T2 (b), T3 (c) and T4 (d).

VFAs composition at peak day and during all the experiments is reported in Fig. 6 a-d. Generally, acetic acid was the dominant species in all experiments (56.4, 37.8, 100 and 47.5 % for T1, T2, T3 and T4 respectively). KMnO_4_ enhanced the production of propionic, iso butyric and butyric acid both in T2 (25.9, 9.9 and 10.9 %) and T4 (35.8, 10.1 and 2.1 %). A remarkable amount of iso valeric acid was produced in T2 (12.2 %) while the remaining acids accounted for less than 4 % altogether in all the tests. Still, T1 showed a much more diversified VFAs composition (19.2, 2.3 and 22.1 % for propionic, iso butyric and butyric acid respectively) than T3 where acetic acid was the only acid detected. Generally, T1-T2 showed a more complex composition than T3-T4. This could be related to a more complex carbohydrates composition and/or a different microbial community composition of the sludges used.

### 3.3 KMnO_4_ addition implications

Results obtained in this study, show that the oxidant addition enhanced the hydrolysis step, thus promoting COD solubilisation (Fig. 2c), and the acidogenic process (Fig. 2a–b). Since an alkaline pH was generated after the oxidant addition, free radicals likely played an important role in the oxidation mechanism [11] during the first fermentation days. Indeed, previous studies [19,39] have demonstrated that oxidants can disrupt the composition of various cell components. However, free radicals oxidation mechanisms are still unclear, since these species are highly reactive and so hard to monitor during the process [40]. Focusing the attention on oxidative free radicals, the unpaired electron in an atomic orbital makes them highly reactive towards electron-rich groups such as amino groups, which are present in proteins structure, or π bond (particularly abundant in recalcitrant organics).

Since no significant differences were found in proteins and carbohydrates (Table 2) concentration between the two sludges used, it can be assumed that recalcitrant organics played a crucial role in the process. Also, KMnO_4_ slightly enhanced COD solubilisation and VFAs production when A plant's sludge was tested (T1 and T2). On the other hand, T4 performance was incredibly better than T3. These results, coupled with the oxidant mechanism, assume that a higher recalcitrant organics concentration was present in plant B's sludge, thus not achieving a feasible VFAs production without KMnO_4_. These results confirm the oxidant positive effect on acidogenic fermentation but, at the same time, suggests that its usage may be beneficial for sludge with high amount of recalcitrant organics and low VSS/TSS ratio, as for plant's B sludge.

## 4 Conclusions

Batch fermentation tests were performed by using sewage sludge as feedstock with the final aim to produce VFAs. The aim was to investigate the influence of an oxidant, KMnO_4_, in sludge acidogenic fermentation by taking into account different sludge's features. Results showed that the pre-treatment enhanced the organic matter solubilisation up to +344 % with a 40 % VSS reduction. VFAs accounted for more than 50 % of sCOD and their composition (mainly acetic and propionic acid) makes KMnO_4_ addition a good pre-treatment to produce a VFAs rich stream suitable for the PHA production process. Still, KMnO_4_ was far more effective when used for a sludge resistant to the acidogenic fermentation process. Future activities will investigate the influence of different pre-treatment, potentially scaling up the experiments in the pilot plant configuration.

## Data availability statement

The data that has been used is confidential.

## CRediT authorship contribution statement

**Antonio Mineo:** Data curation, Formal analysis, Writing – original draft, Writing – review & editing. **Alida Cosenza:** Data curation, Methodology, Visualization, Writing – original draft, Writing – review & editing. **Bing-Jie Ni:** Conceptualization, Writing – original draft, Writing – review & editing. **Giorgio Mannina:** Conceptualization, Data curation, Project administration, Resources, Supervision, Writing – original draft, Writing – review & editing.

## Declaration of competing interest

The authors declare that they have no known competing financial interests or personal relationships that could have appeared to influence the work reported in this paper.
